# Fibre crosslinking drives the emergence of order in a three-dimensional dynamical network model

**DOI:** 10.1098/rsos.231456

**Published:** 2024-01-31

**Authors:** Pauline Chassonnery, Jenny Paupert, Anne Lorsignol, Childérick Séverac, Marielle Ousset, Pierre Degond, Louis Casteilla, Diane Peurichard

**Affiliations:** ^1^ RESTORE, Université de Toulouse, Inserm U1031, EFS, INP-ENVT, UPS, CNRS ERL5311, Toulouse, France; ^2^ Inria Paris, team MAMBA, Sorbonne Université, CNRS, Université de Paris, Laboratoire Jacques-Louis Lions UMR7598, 75005 Paris, France; ^3^ Institut de Mathématiques de Toulouse, UMR5219, Université de Toulouse, CNRS, UPS, 31062 Toulouse Cedex 9, France

**Keywords:** interaction networks, three-dimensional mathematical modelling, self-organization, extracellular matrix, dynamical crosslinking, architecture emergence

## Abstract

The extracellular-matrix (ECM) is a complex interconnected three-dimensional network that provides structural support for the cells and tissues and defines organ architecture as key for their healthy functioning. However, the intimate mechanisms by which ECM acquire their three-dimensional architecture are still largely unknown. In this paper, we study this question by means of a simple three-dimensional individual based model of interacting fibres able to spontaneously crosslink or unlink to each other and align at the crosslinks. We show that such systems are able to spontaneously generate different types of architectures. We provide a thorough analysis of the emerging structures by an exhaustive parametric analysis and the use of appropriate visualization tools and quantifiers in three dimensions. The most striking result is that the emergence of ordered structures can be fully explained by a single emerging variable: the number of links per fibre in the network. If validated on real tissues, this simple variable could become an important putative target to control and predict the structuring of biological tissues, to suggest possible new therapeutic strategies to restore tissue functions after disruption, and to help in the development of collagen-based scaffolds for tissue engineering. Moreover, the model reveals that the emergence of architecture is a spatially homogeneous process following a unique evolutionary path, and highlights the essential role of dynamical crosslinking in tissue structuring.

## 1. Introduction

The adequate architecture of any organ is mandatory for their efficient physiological function and any changes are associated with function impairment and putative developing dysfunctions and diseases [1–3]. All biological tissues contain scaffolds of non-cellular components called extracellular matrices (ECMs) [4]. Despite the great variability of proteins that make up the ECM (macromolecules such as collagen, glycoproteins etc.), it can be seen as a dynamic physical network of fibres interconnected by molecular bonds, i.e. crosslinks, generating a connected and elastic environment for the surrounding cells [2].

The network structure is in a state of constant remodelling, which is crucial to maintain tissue integrity and function. Crosslinks, however, can unbind spontaneously or under tension, which leads to viscoplastic material responses, such as softening and tension relaxation [5]. Fibrosis and ageing are also characterized by an increase of enzymatic and non-enzymatic crosslinks [6,7] and this increase in crosslinking prevents ECM degradation by matrix metalloproteinases, both events leading to a decrease of ECM remodelling [8]. Altogether, these events induce greater stiffness and the arrangement of the collagen fibres becomes less organized and more loose and fragmented, hence weakening tissue integrity and strength [9,10]. An understanding of the basic organizing principles of ECM structure in three dimensions also helps in apprehending the complex dynamics of pathological tissues from degenerative diseases or tumour [8].

Because the global architecture of fibre networks seems to be fundamental for controlling tissue functions, modelling the process of ECM structure emergence will greatly improve our understanding of tissue biology and plasticity in physiological or pathological conditions. Numerous models of fibre networks can be found in the literature. Due to their simplicity and flexibility, the most widely used models are individual based models (IBM), which describe the behaviour of each agent (e.g. a fibre element) and its interactions with the surrounding agents over time [11,12]. However, IBMs have a high computational cost which can become intractable when studying systems composed of too many agents, or systems at large scales, either spatial or temporal. In such cases, continuous or mean-field kinetic models may be preferred [13–17] since they are less costly, but at the expense of a loss of information at the individual level. Since it is well acknowledged that microstructure configurations modulate the macroscopic properties of crosslinked fibre networks [18], preserving the microscopic level description is of great importance to model tissue emergence.

Most of the computational models developed thus far for mimicking ECM networks are two-dimensional [14,16,19–27]. Few studies have been conducted on three-dimensional models [28–35], although these are expected to yield different, more realistic results than two-dimensional ones since they better mimic biological structures themselves immersed in three-dimensional environments. One of the reasons for fewer three-dimensional models is the great increase in the number of agents needed to achieve a given spatial density and thus in the associated computational cost. Another reason is the lack of high quality data on ECM organization in three dimensions. However, the latter is becoming less and less of an issue with recent improvements in high resolution three-dimensional imaging and its availability. Among existing three-dimensional models, few of them feature dynamical crosslinking of ECM components. In [30,32,36], various models of three-dimensional fibrous networks composed of permanent or transient crosslinks (remodelling) are proposed. However, most of these models feature ECM remodelling in reaction to external factors (applied load [30,37], migrating cells [32], contractile cells [36]), and the literature so far provides few cues on the mechanisms underlying fibre self-organization.

In the present paper, we test the hypothesis that fibre macrostructures could spontaneously emerge without appealing to contact guidance or external mechanical challenges, as a result of simple mechanical interactions between the fibre elements composing the ECM network. We assess this hypothesis by means of a simple three-dimensional model where ECM fibres are discretized into unit fibre elements, consisting of non-stretching and nonflexible spherocylinders with the ability to spontaneously link to and unlink from their close neighbours. This dynamical crosslinking mechanism allows us to model both the overall temporal plasticity of the network and the complex physical properties of biological fibres such as elongation, bending, branching and growth, thus compensating our minimalistic description of the fibre units. We stress that in this paper, rather than developing a very complex model to reproduce the whole complexity of real tissues (at the cost of losing explicability), a large part of which corresponds to the redundancy of mechanisms to ensure the robustness of structures and regulations, we aim to keep a mathematical framework as simple as possible in order to break the complexity and shed light on some main and basic components at play in the emergence of fibrous structures. The relevance of such an approach was previously validated in the frame of adipose tissue morphogenesis and regeneration in two dimensions [27,38].

Through computational simulations and exhaustive parametric analysis, we demonstrate that organized macrostructures can spontaneously emerge without external guidance. Overall, this study provides a comprehensive view on the role of ECM connectivity on tissue architecture emergence:

— The model reveals that tissue architecture at equilibrium is simply controlled by the number of crosslinks per fibre in the network, an emerging variable not directly linked to the model parameters. If further validated on real tissues, this simple emerging variable could become an important putative target to control and predict the development of the architecture of biological tissues. Because of its simplicity, this variable is amenable to experimental measurements and could represent a major target for the development of therapeutic drugs to induce tissue recovery after injury, prevent tissue degradation during ageing, or help in the design of engineering collagen scaffolds for tissue regeneration.
— A deep exploration of the model parameters reveals that this emerging variable, and therefore the global organization abilities of tissues, depend on a complex interplay between the model parameters related to the crosslinks, i.e their remodelling speed and their linked fibre fraction. These results rationalize how even subtle changes in fibre networks dynamical crosslinking can drive tissue reorganization and suggest that the development of biological crosslinkers to control ECM connectivity as a target for tissue reconstruction must carefully account for different parameters such as tissue remodelling activities.
— Finally, a temporal analysis of the model simulations reveals that the different tissue architectures follow a simple and unique evolutionary path on timescales controlled by their remodelling characteristics, providing new insights into the temporal evolution of tissue structures as a function of the ECM remodelling properties.

## 2. Models and methods

### 2.1. Description of the model

The three-dimensional ECM is discretized into unit fibre elements consisting of line segments of fixed and uniform length, represented by their centres and directional unit vectors. We consider the following biological and mechanical features: (*i) Fibre resistance to pressure:* We suppose that fibre elements repel each other at short distances, which models size-exclusion effects. This is achieved via a repulsive force between close fibres based on Hertzian theory [39]. This amounts to model fibres as spherocylinders of a given radius and length, that can interpenetrate each other. The intensity of the repulsion force *α*_rep_ controls the amount of overlapping between fibres. (*ii) Fibre elongation and breakage:* In addition to carrying a unit of ECM fibre strength, fibre elements also carry a unit of fibre length. However, we provide a way to create longer fibres by allowing two nearby fibres to form a link. A crosslink is modelled as a linear spring with a given spring stiffness, connecting the two closest points of the fibre pair at the time of its creation. There is no prescription for the location of the crosslinks along the body of the fibres they connect. Several consecutively cross-linked fibre elements would model a long and flexible fibre having the ability to adopt complex geometries. Therefore, the cross-linking process models fibre elongation [40]. The stiffness constant of the springs *α*_rest_ controls the possible extension of the long fibres. Symmetrically, pairs of cross-linked fibres can spontaneously unlink, allowing for fibre breakage describing ECM remodelling processes [41]. Linking and unlinking processes follow Poisson processes with frequencies *ν*_link_ and *ν*_unlink_, respectively. As a result, the linked fibre ratio $\chi_{\mathrm{link}}=\nu_{\mathrm{link}}/(\nu_{\mathrm{link}}+\nu_{\mathrm{unlink}})$ represents the equilibrium fraction of linked fibres among the pairs of neighbouring fibres. (*iii) Crosslink fibre alignment:* To model the ability of long fibres (those made of several cross-linked fibre units) to offer a certain resistance to bending, linked fibres are subjected to a potential torque at their junction. This torque vanishes when the fibres are aligned, and consequently acts as a linked-fibre alignment mechanism. This torque is characterized by a stiffness parameter *α*_align_ playing the role of a flexural modulus. (*iv) Large friction regime:* As the Reynolds number in most biological tissues is very small [42], we suppose that inertial forces can be neglected and we consider an over-damped regime for fibre motion and rotation.

Each of the mechanical interactions due to fibre-fibre repulsion (i), fibre-fibre attachment due to crosslinks (ii) and crosslinked fibre-fibre alignment (iii) generate elementary forces and torques between fibre pairs. The total force (resp. torque) acting on a fibre is then computed as the sum of all the elementary forces (resp. torques) generated by the elements interacting with this fibre. The motion and rotation of the fibre is then deduced from Newton’s equation of motion in an over-damped regime. More specifically, the *N*_fib_ fibre elements are represented by straight lines of fixed length *L*_fib_ represented by their centres $X_{k}(t)\in \Omega \subset R^{3}$ and their non-oriented directional unit vectors $\omega_{k}(t)\in S_{2}^{+}$. Moreover, from the fibre-fibre repulsion interaction, fibres may be seen as soft spherocylinders of radius *R*_fib_. We denote by (*p*_*k*,*m*_(*t*))_*k*,*m*_ the fibre connectivity matrix, that is *p*_*k*,*m*_(*t*) is equal to 1 if fibres *k* and *m* are linked at time *t* and to 0 otherwise.

The motion and rotation of fibre *k* are then given by

2.1 $$\begin{array}{c} \begin{array}{c} \mu_{\mathrm{fib}}L_{\mathrm{fib}}\frac{dX_{k}}{dt}(t)=\sum_{m=1}^{N_{\mathrm{fib}}}(F_{k,m}^{\mathrm{rep}}(t)+p_{k,m}(t)F_{k,m}^{\mathrm{rest}}(t))\;\;\\ \mu_{\mathrm{fib}}L_{\mathrm{fib}}^{3}\frac{d\omega_{k}}{dt}(t)=\sum_{m=1}^{N_{\mathrm{fib}}}(T_{k,m}^{\mathrm{rep}}(t)+p_{k,m}(t)(T_{k,m}^{\mathrm{rest}}(t)+T_{k,m}^{\mathrm{align}}(t)))\wedge \omega_{k}(t) \end{array}\;\forall k\in [[1,N_{\mathrm{fib}}]], \end{array}\}$$

where $F_{k,m}^{\mathrm{rep}}(t)$ and $T_{k,m}^{\mathrm{rep}}(t)$ are the force and torque associated with the repulsion between fibres *k* and *m*, $F_{k,m}^{\mathrm{rest}}(t)$ and $T_{k,m}^{\mathrm{rest}}(t)$ are the force and torque due to the presence of a spring (crosslink) connecting fibres *k* and *m*, and $T_{k,m}^{\mathrm{align}}(t)$ is the alignment torque generated by this crosslink. We refer to appendix A.1 for the detailed computations of these forces and torques.

### 2.2. Description of the simulation set-up and biological relevance of the model parameters

The spatial domain Ω is a cuboid of side lengths *L*_*x*_, *L*_*y*_ and *L*_*z*_, respectively, in the *x*, *y* and *z*-dimension, centred on the origin

$$\Omega =\left[-\frac{L_{x}}{2},\frac{L_{x}}{2}\right]\times \left[-\frac{L_{y}}{2},\frac{L_{y}}{2}\right]\times \left[-\frac{L_{z}}{2},\frac{L_{z}}{2}\right].$$

For the sake of simplicity, we assume periodic boundary conditions: an agent exiting the domain by one side re-enters immediately from the opposite side, and interactions between agents are computed using the periodicized Euclidean distance. Fibres are initially randomly inseminated inside the domain according to a uniform law for both position and orientation. The differential system (2.1) is then numerically solved using a discrete upwind Euler scheme with adaptive time step, which has a very low computational cost. Details of the numerical implementation are given in appendix A.2.

The physical scaling of all the parameters of the model, as well as the values used in the simulations, are described in table 1. A few points may be noted: (a) the perception distance for link creation $d_{\mathrm{link}}^{\max}$ and the link unloaded length $d_{\mathrm{link}}^{\mathrm{eq}}$ are both equal to the diameter of a fibre 2 *R*_fib_. This means that the fibre units (spherocylinders) connect with the fibres they are in contact with or closer, and that the link tries to keep the bodies of the spherocylinders touching. In this regime, the presence of the links therefore participate in a non-overlapping configuration of the fibres. (b) The size of the domain is approximately four times the size of a fibre along its main axis (numerical checks were made by-hand to select a size of domain which optimizes between computation time and boundary effects) and (c) the fibre aspect-ratio *L*_fib_/2 *R*_fib_ = 6 is quite small compared to the values used in other models of the ECM, which usually varies between 250 and 10^4^ [15,33,34]. This compensates for the fact that these models directly account for fibre bending and/or fibre elongation, while our long fibres correspond to a sequence of crosslinked fibre units. On the same note, we stress the fact that our fibre units do not aim at modelling the individual collagen fibrils making up collagen fibres in ECM, but rather correspond to an intermediate scale where one fibre unit of our model is already a set of twined collagen fibrils that run in parallel to form a larger bundle [43].

**Table 1.** Model parameters.

name	value	units	description
agents		
*N*_fib_	[1500, 3000]	n.a.	number of fibres
*L*_fib_	6	*L*	fibre length
*R*_fib_	0.5	*L*	fibre radius
mechanical interactions		
*α*_rep_	12.5	*M* · *L*^−1^ · *T*^−2^	magnitude of the repulsion force
*α*_rest_	5.0	*M* · *T*^−2^	magnitude of the elastic restoring force
*α*_align_	2.0	*M* · *L*^2^ · *T*^−2^	magnitude of the alignment torque
$d_{\mathrm{link}}^{\max}$	1.0	*L*	perception distance for link creation
$d_{\mathrm{link}}^{\mathrm{eq}}$	1.0	*L*	link equilibrium length
biological phenomena		
*ν*_link_	[0, 10]	*T*^−1^	network remodelling speed
*χ*_link_	[0.1, 0.9]	n.a.	equilibrium linked fibre fraction
numerical parameters		
*L*_*x*_ = *L*_*y*_ = *L*_*z*_	30	*L*	side length of the cubic domain
*T*_final_	5.10^4^	*T*	total time of simulation

We denote by *ϕ*_fib_ the fibre density of the network, that is the ratio between the total volume of fibres (without overlapping) and the volume of the spatial domain:

2.2 $$\phi_{\mathrm{fib}}=\frac{N_{\mathrm{fib}}V_{\mathrm{fib}}}{\left|\Omega \right|}=N_{\mathrm{fib}}\frac{\pi R_{\mathrm{fib}}^{2}L_{\mathrm{fib}}+(4/3)\pi R_{\mathrm{fib}}^{3}}{L_{x}L_{y}L_{z}}.$$

The quantity *ϕ*_fib_ can be compared to the packing density, that is the maximal fraction of the domain that can be occupied by densely packed fibres. In the case of an ordered packing, the packing density of spherocylinders is *ϕ*_order_ = 0.89, while for random or amorphous packing of spherocylinders with an aspect ratio of 6, the maximal random packing density *ϕ*_random_ ≈ 0.4 [20]. Thus, we may say that a system is ‘sparse’ if its fibre density is below *ϕ*_random_, ‘dense’ if it is between *ϕ*_random_ and *ϕ*_order_, and ‘hyperdense’ if it is above *ϕ*_order_. In the following, we will study two types of systems: dense systems containing *N*_fib_ = 3000 fibres (*ϕ*_fib_ = 0.58) and sparse systems with *N*_fib_ = 1500 fibres (*ϕ*_fib_ = 0.29, corresponding to measurements of extracellular volume fraction in muscle or myocardial fibrosis, see e.g. [44,45]).

For each of the three types of mechanical forces in the system, we define the ‘characteristic interaction time’ as the time needed for two isolated fibres interacting only via this force and initially positioned in the most unfavourable configuration to reach 99% of the equilibrium state. For repulsion, *T*_rep_ is the time needed for two fully overlapped fibres (**X**_1_ = **X**_2_ and *ω*_1_ = *ω*_2_) to move apart by 99% of their equilibrium distance 2 *R*_fib_ (i.e. ‖**X**_1_ − **X**_2_‖ = 0.99 × 2 *R*_fib_). Similarly, for the elastic spring *T*_rest_ is the time needed for two fibres that are initially fully overlapping and crosslinked at their centre to move apart by 99% of their equilibrium distance $d_{\mathrm{link}}^{\mathrm{eq}}$. On the other hand, for nematic alignment *T*_align_ is the time needed for two perpendicularly intersecting fibres (**X**_1_ = **X**_2_ and *ω*_1_⊥*ω*_2_) crosslinked at their centre to reach a relative angle $\arccos(\omega_{1}\cdot \omega_{2})=0.9^{\circ }$.

Explicit computation leads to the following formula (numerical values are given for the parameters presented in table 1)

2.3 $$\begin{array}{cc} & T_{\mathrm{rep}}=\frac{27\mu_{\mathrm{fib}}L_{\mathrm{fib}}}{4\sqrt{2}\;R_{\mathrm{fib}}\;\alpha_{\mathrm{rep}}}=4.32\;U_{t},\\ & T_{\mathrm{rest}}=\ln(10)\frac{\mu_{\mathrm{fib}}L_{\mathrm{fib}}}{\alpha_{\mathrm{rest}}}=2.76\;U_{t}\\ \;\mathrm{and}\; & T_{\mathrm{align}}=4.27\frac{\mu_{\mathrm{fib}}L_{\mathrm{fib}}^{3}}{\alpha_{\mathrm{align}}}=462\;U_{t}. \end{array}\}$$

It may be noted that the alignment interaction is much slower than the repulsive and elastic restoring forces. In this regime, fibre elements are quite rigid and connected by strong springs (crosslinks), enabling us to prevent local accumulation of fibres and overstretching of long fibres (those made of several crosslinked fibre units).

## 3. Results

### 3.1. Matrix crosslinking drives the local alignment of three-dimensional dynamical fibre networks

**Figure 1.**
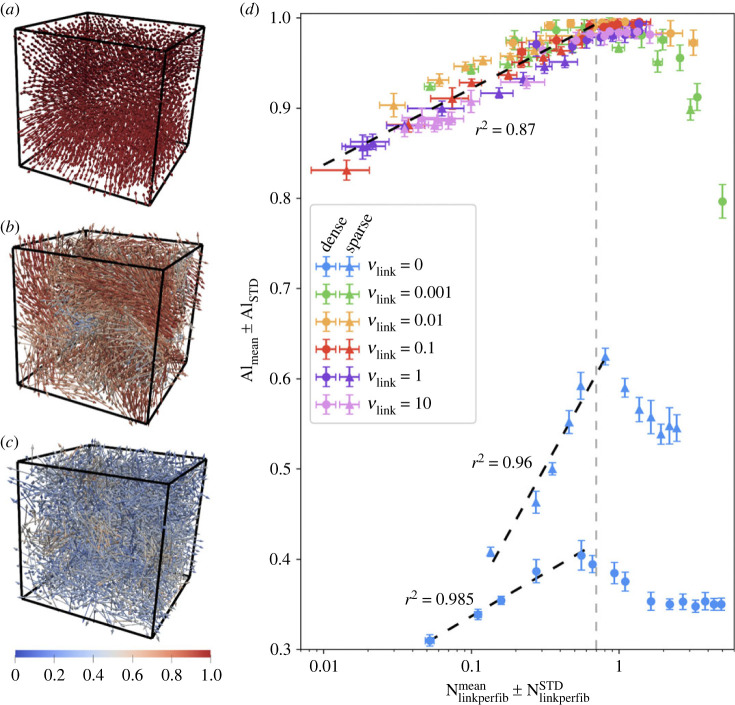
(*a–c*) Illustration of the various structures that can be observed at equilibrium. Fibres are represented by double-headed arrows and coloured according to their local alignment with their neighbours (from blue: Al_*k*_ = 0 to red: Al_*k*_ = 1). The structures range from systems with uniformly high local alignment indicator (*a*) through systems with heterogeneous, intermediate local alignment indicator (*b*) to disordered systems with uniformly low local alignment indicator (*c*). (*d*) Value of Al_mean_ according to $N_{\mathrm{linkperfib}}^{\mathrm{mean}}$ at equilibrium, with colour depending on the remodelling speed *ν*_link_ and horizontal and vertical error-bars indicating the standard deviation $N_{\mathrm{linkperfib}}^{\mathrm{STD}}$ and Al_STD_, respectively. The grey dashed-line indicates the critical value of $N_{\mathrm{linkperfib}}^{\mathrm{mean}}$ and the black dashed lines the three logarithmic fits obtained for $N_{\mathrm{linkperfib}}^{\mathrm{mean}}<N_{\mathrm{critic}}$.

In figure 1*a*–*c*, we show various structures that can be obtained with our model by playing on the parameters in the ranges indicated in table 1. The fibres are represented by double arrows and coloured as a function of their local alignment with their neighbours. We refer the readers to appendix B.1 for more details on the computation of this quantifier, and just mention that the local alignment of fibre *k*, denoted Al_*k*_, is equal to 1 (fibre coloured in red) if all the neighbouring fibres display the exact same direction as fibre *k*, and to 0 (fibre coloured in blue) if the neighbouring fibres display uniformly distributed directional vectors. Moreover, we show in appendix B.1 that this quantifier is able to discriminate between fibres located in randomly oriented environments (corresponding to Al_*k*_ < 0.5), fibres located in nearly planar environments (leading to Al_*k*_ around 0.7), and fibres located in nearly uni-directional environments (leading to Al_*k*_ above 0.8).

As one can observe, the fibre structures obtained at equilibrium range from highly aligned systems (mainly composed of red fibres, see figure 1*a*) to disordered systems with a low local alignment (mainly composed of fibres coloured in blue, see figure 1*c*). The model can also produce intermediate states composed of fibres with a median local alignment (figure 1*b*).

In order to assess the alignment states of our different fibre networks, we computed the mean of the local alignment indicator Al_*k*_ over all the fibres of the system, denoted by Al_sim_. To account for stochastic variability (due to the random initial condition and the stochastic linking and unlinking processes), we computed the mean and standard deviation of Al_sim_ over 10 simulations conducted with the same set of parameters, denoted by Al_mean_ and Al_STD_. Similarly, we denote by *N*_linkperfib_ = *N*_links_/*N*_fib_ the number of links per fibres in a network and by $N_{\mathrm{linkperfib}}^{\mathrm{mean}}$ and $N_{\mathrm{linkperfib}}^{\mathrm{STD}}$ its average and standard deviation over 10 simulations. We stress the fact that *N*_linkperfib_ = 0.5 if all the fibres are connected to a neighbouring fibre.

By plotting the alignment quantifier Al_mean_ as a function of the number of links per fibre $N_{\mathrm{linkperfib}}^{\mathrm{mean}}$ (both computed on the systems at equilibrium), we discovered a striking and major correlation between these two quantities. This correlation is shown in figure 1*b*, with horizontal and vertical error-bars indicating the inter-simulation standard deviations $N_{\mathrm{linkperfib}}^{\mathrm{STD}}$ and Al_STD_, respectively. The different markers indicate different fibre densities (dots for dense systems and triangles for sparse ones), the different colours refer to different networks dynamics *ν*_link_, and inside each colour series *χ*_link_ is increasing with $N_{\mathrm{linkperfib}}^{\mathrm{mean}}$.

Figure 1*d* reveals that the values of Al_mean_ and $N_{\mathrm{linkperfib}}^{\mathrm{mean}}$ at equilibrium are highly correlated. When $N_{\mathrm{linkperfib}}^{\mathrm{mean}}$ is inferior to a critical threshold *N*_critic_ ≈ 0.7 (indicated with a grey dashed line on figure 1*d*), there is a logarithmic correlation between the number of links per fibre in the network and its mean alignment indicator (black dashed lines in figure 1*d*)

3.1 $${\mathrm{Al}}_{\mathrm{mean}}\approx \alpha \log(N_{\mathrm{linkperfib}}^{\mathrm{mean}})+\beta ,$$

with

— *α* = 0.037, *β* = 1.006 and coefficient of determination *r*^2^ = 0.87 for dynamical systems (non-blue markers);
— *α* = 0.129, *β* = 0.651 and coefficient of determination *r*^2^ = 0.96 for sparse non-dynamical networks (blue triangles);
— *α* = 0.042, *β* = 0.433 and coefficient of determination *r*^2^ = 0.985 for dense non-dynamical networks (blue dots).

Then, when $N_{\mathrm{linkperfib}}^{\mathrm{mean}}>N_{\mathrm{critic}}$ we observe an abrupt drop of the equilibrium value of Al_mean_. Surprisingly and very interestingly, for dynamical systems (*ν*_link_ > 0) there is no difference in alignment induced by the fibre density or the link characteristics *ν*_link_ and *χ*_link_: the correlation observed is the same for all sets of points.

The second major observation from figure 1*d* is the difference between non-dynamical and dynamical networks at equilibrium. Indeed non-dynamical networks, composed of a fixed number of links, are systematically less aligned than dynamical ones (compare the values of Al_mean_ between the blue markers and the other colours). Moreover, although we do recover the same type of correlation between the fibre local alignment and the number of links per fibre in the network, for non-dynamical networks this correlation significantly depends on the fibre density. However, the critical number of links *N*_critic_ allowing for larger alignment is the same for non-dynamical networks, either dense or sparse, and for dynamical networks. Therefore, *N*_critic_ seems to be a general critical network connectivity value controlling the local alignment abilities of various networks.

Altogether, these results show that the emergence of organized networks (i) requires some remodelling abilities of the ECM matrix and (ii) is mainly controlled by the number of links per fibre.

### 3.2. ECM architecture emergence is driven by a complex interplay between remodelling speed and linked fibre fraction

The previous section took a particular focus on the local arrangement of the fibre units composing our three-dimensional fibre network, with little information on the global structures at the population scale. In this section, we aimed to characterize quantitatively the macrostructures that emerge in our networks. To this end, we used the stereographic projection of the fibre directional vectors. Disregarding the spatial position of a fibre, we represented its directional vector as a point on the surface of the unit half-sphere in three dimensions and then projected it onto the unit disk in two dimensions (see appendix B.2 for a detailed explanation).

**Figure 2.**
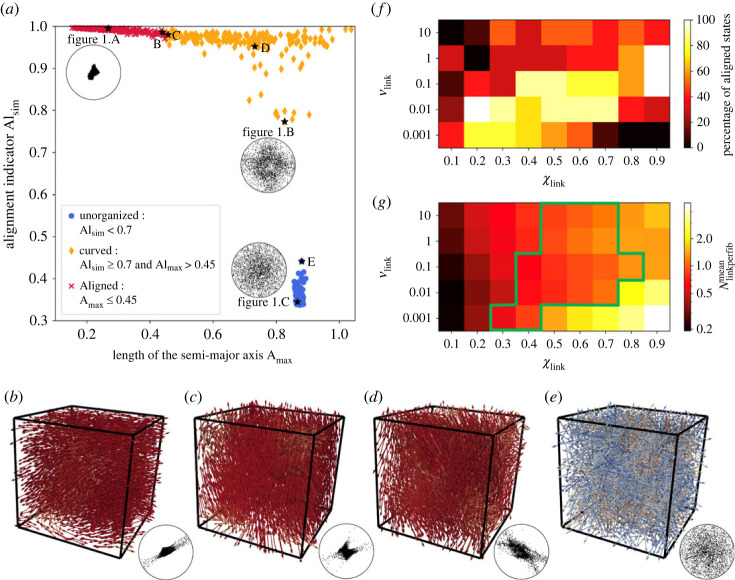
(*a*) Alignment indicator Al_sim_ versus semi-major axis length of the covariance ellipse of the stereographic projection A_max_, for each simulation of dense systems (*N*_fib_ = 3000). Red crosses correspond to systems in an aligned state, orange diamonds to curved states and blue dots to unorganized states. The simulations previously displayed in figure 1 are indicated with a black star and their stereographic projection given as inset. Panel (*b*–*e*) displays the equilibrium state of a few other simulations, whose positions on the diagram are also indicated with a black star. (*f*) Heatmap of the percentage of simulations ending in an aligned state (versus a curved state), for dynamical dense networks as function of the values of the network remodelling speed *ν*_link_ (in ordinate) and the equilibrium linked fibre fraction *χ*_link_ (in abscissa). (*g*) Heatmap of the number of links per fibre $N_{\mathrm{linkperfib}}^{\mathrm{mean}}$ for dynamical dense networks as function of the values of the network remodelling speed *ν*_link_ (in ordinate) and the equilibrium linked fibre fraction *χ*_link_ (in abscissa). Zones where $N_{\mathrm{linkperfib}}^{\mathrm{mean}}\approx N_{\mathrm{critic}}$ have been highlighted in green.

As shown in figure 2, this representation enabled us to characterize the different global organizations of our fibre networks. Indeed, we observed three different types of stereographic projections in our simulations: fibres' directional vectors very concentrated around the centre of the disc, corresponding to a global alignment of the system (figure 1*a*, with stereographic projection shown as inset in figure 2*a*), fibres' directional vectors homogeneously distributed on the disc corresponding to a global disorder (figures 1*c* and 2*e*), and fibres' directional vectors distributed along a preferential axis, with complete depletion in the direction perpendicular to this axis, corresponding to global curved/plane structures (figure 2*b*–*d*).

Together with the local alignment quantifier Al_sim_, we were now able to quantitatively characterize the different local and global fibre organizations inside our networks. We considered a system to be locally aligned if Al_sim_ was above 0.7 (see appendix B.1 for justification of this value). At the same time, we considered that a system was globally aligned if its stereographic projection covariance ellipse had a semi-major axis smaller than 0.45 (implying that the point cloud covers less than 20% of the whole projection disk).

We therefore classified the simulations outcomes into three different states (unorganized, curved and aligned) using table 2. We ran a total of 1080 numerical simulations, exploring various values of the parameters *ν*_link_, *χ*_link_ and *N*_fib_ in the broad ranges indicated in table 1, and counted among their outcomes:

— 180 unorganized states (all occurring in non-dynamical systems, i.e. *ν*_link_ = 0),
— 661 curved states,
— 239 aligned states (among which only 12 occurred in sparse systems).

**Table 2.** Classification of the simulations outcomes into different states based on the local quantifier Al_sim_ and the global quantifier A_max_. The case $\left\{{\mathrm{Al}}_{\mathrm{sim}}<0.7\;\&\;A_{\max}\leq 0.45\right\}$ never occurs in our simulations and is thus unnamed.

A_max_			
		≤0.45	>0.45
Al_sim_	≥0.7	**aligned state:** alignment both local and global	**curved state:** alignment local but not global
	<0.7	(alignment global but not local)	**unorganized state:** no alignment, either local or global

Figure 2*a* shows the equilibrium values of quantifiers Al_sim_ and A_max_ for dense systems (see electronic supplementary material, appendix C.2 for the equivalent figure on sparse systems). The points are coloured according to the states defined previously (blue dots correspond to unorganized states, orange diamonds to curved states and red crosses to aligned states). The simulations already displayed in figure 1 are indicated with a black star and their stereographic projection shown as inset. Four other simulation outcomes are singled out with black stars on the phase diagram and illustrated with a three-dimensional view and stereographic projection in the *b* to *e*.

From figure 2*a*, we first observe that the unorganized states (blue dots) form a small, compact group of points with large semi-major axis length, while the aligned states (red crosses) make a long thin group with very high alignment indicator. On the other hand, the curved states (orange diamonds) form a scattered cloud of points with a broad range of values for both the semi-major axis length and the alignment indicator. Moreover, we observe that the transition between unorganized and curved states is very sharp (note the gap between the blue dots and orange diamonds in *a*). Indeed, no simulation displays an average alignment indicator at equilibrium between 0.65 and 0.77 (including sparse systems, see electronic supplementary material, appendix C.2), and there is a marked difference between the least organized of the curved states (illustrated in figure 1*a*) and the most organized of the unorganized states (illustrated in figure 2*e*). This confirms our choice of 0.7 for the threshold value between unorganized and curved states.

On the contrary, the transition from curved to aligned states is not a clear switch but a continuum of structures that can be illustrated by the two borderline cases in figure 2*b*,*c*. Thus, one must be aware that the partition between curved and aligned states is partly arbitrary and depends on the choice of the threshold. However, this classification into three states allowed us to distinguish between unorganized networks, globally aligned networks and networks locally aligned with twisting capacities at the population level, enabling us to go deeper into the model parameters controlling tissue architecture emergence at different scales.

We first found that the sharp transition between unorganized and curved states was fully controlled by the remodelling speed of the network *ν*_link_. Indeed, unorganized states were only and systematically observed for non-dynamical networks (*ν*_link_ = 0), while dynamical networks (*ν*_link_ > 0) never equilibrated in unorganized states but self-organized into either curved or aligned states, and this independently on the fibre density of the network (see electronic supplementary material, appendix C.2). By contrast, the transition between curved and aligned states is not controlled by a unique model parameter but is the interplay between several parameters.

Indeed, figure 2*f* shows a heatmap of the percentage of simulations ending in an aligned state (versus a curved state), for dynamical dense networks (see electronic supplementary material, appendix C.2 for results on sparse networks), depending on the values of the network remodelling speed *ν*_link_ (in ordinate) and the equilibrium linked fibre fraction *χ*_link_ (in abscissa). As one can observe in figure 2*f*, there is a nonlinear relationship between the global alignment capacities of the networks and the parameters *ν*_link_ and *χ*_link_. Indeed, analysis of the heatmap reveals that (i) reduced linked fibre fraction *χ*_link_ can increase global alignment outputs because, for low-remodelling networks, the formation of crowded interconnected fibre structures inhibiting fibre motion is relieved by reduced link density. Moreover, (ii) the global alignment of networks with intermediate remodelling rates may undergo little change with reduced linked fibre-fraction and (iii) the global alignment ability of fast-remodelling networks will likely be impaired by reduced linked fibre-fraction. These results show that the different types of tissue architectures (aligned, curved or unorganized) depend on an interplay between parameters *ν*_link_ and *χ*_link_. While ECM local alignment can be explained by the simple emerging variable that is the number of links per fibre in the network (as shown in §3.1), its direct relation with model parameters *N*_fib_, *ν*_link_ and *χ*_link_ is more complex. Indeed, figure 2*g* shows a heatmap of the number of links per fibre in the network $N_{\mathrm{linkperfib}}^{\mathrm{mean}}$ as a function of *ν*_link_ and *χ*_link_ for dense dynamical networks (same simulations as *f*). It demonstrates that $N_{\mathrm{linkperfib}}^{\mathrm{mean}}$ is indeed an emerging variable, in the sense that it is not directly linked to the parameters *ν*_link_ and *χ*_link_ but rather is the result of a complex interplay between the two. Indeed, the number of links per fibre in the network increases along the diagonal, as *ν*_link_ decreases and *χ*_link_ increases (from top left to bottom right corner of the heatmap), crossing the critical threshold *N*_critic_ doing so (the cells where $N_{\mathrm{linkperfib}}^{\mathrm{mean}}\approx N_{\mathrm{critic}}$ are framed in green in *g*). These results explain why the proportion of aligned structures are maximal along the diagonal from the bottom left to the top right (i.e broadly perpendicular to the gradient of the emergent parameter). These results extend to the case of sparse networks (see electronic supplementary material, fig. 8 in appendix C.2), confirming the strong correlation between ECM alignment abilities and the number of links per fibre they contain.

These results show that our networks can be seen as corresponding to different phases of physical materials depending on their remodelling abilities. If non-dynamical networks can be seen as solid structures unable to spontaneously reorganize, dynamical networks have properties reminiscent of fluid materials, the global architecture of which being controlled by an interplay between their remodelling speed and their linked fibre fraction. In the next section, we study the evolution in time of the structures, enabling us to give more insights into the role of these parameters in tissue structuring in time.

### 3.3. ECM architecture emergence follows a unique evolutionary path on timescales controlled by their remodelling characteristics

In this section, we study the temporal evolution of the spatial structures. Our very first observation is that, for all sets of parameters, the evolution in time of the quantifier Al_mean_ follows a logarithmic growth (see electronic supplementary material, appendix C.4 for more details). We will use as a time reference the time-constant of this growth, denoted *τ*_Al_, which corresponds to the time needed for the quantifier to reach 63% of its asymptotic value (Al_mean_ ≈ 0.7 in our case).

**Figure 3.**
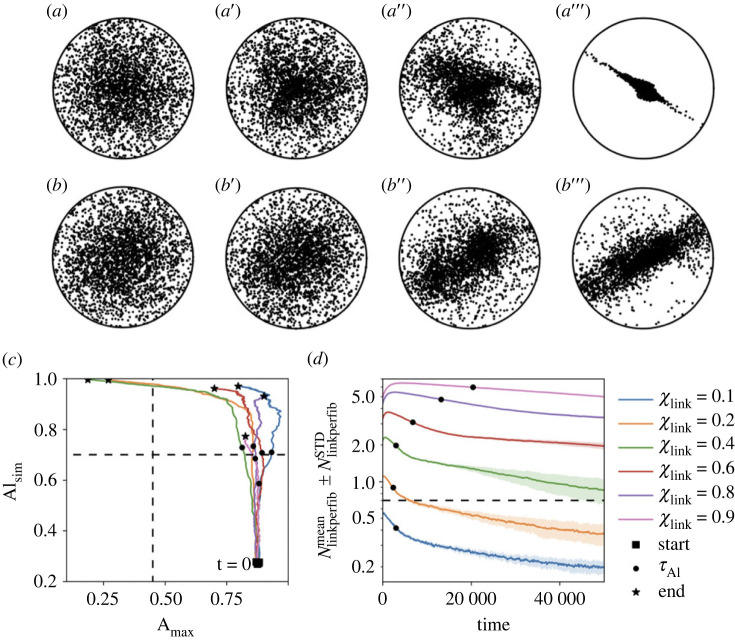
Temporal evolution of dense systems (*N*_fib_ = 3000) with various linking dynamics. *Panels *a*-*a*”’:* Stereographic projection of the system at times 0.5*τ*_Al_ (*a*), *τ*_Al_ (*a’*), 3*τ*_Al_ (*a*”) and *T*_final_ (*a*”’), for one simulation with *ν*_link_ = 0.1 and *χ*_link_ = 0.8. *Panels b–b”’:* Stereographic projection of the system at times 0.5*τ*_Al_ (*b*), *τ*_Al_ (*B’*), 3*τ*_Al_ (**b*”*) and *T*_final_ (*b”’*), for one simulation with *ν*_link_ = 0.001 and *χ*_link_ = 0.8. (*c*) Trajectory in the phase plane A_max_ versus Al_sim_ of individual simulations for slow-remodelling dense networks *ν*_link_ = 0.001 and various linked fibre fractions *χ*_link_. The initial position is indicated with a black square, the final position with a black star and the time-constant *τ*_Al_ with a black circle. The limits between each class of structures are drawn in dashed lines. (*d*) Evolution of $N_{\mathrm{linkperfib}}^{\mathrm{mean}}$ for slow-remodelling dense networks *ν*_link_ = 0.001 and various linked fibre fractions *χ*_link_, with shading indicating the inter-simulation standard deviation $N_{\mathrm{linkperfib}}^{\mathrm{STD}}$. The critical value *N*_critic_ is indicated with a dashed line and the time-constant *τ*_Al_ with a black circle.

Movies displaying the full temporal evolution of a few simulations are available in supplementary data (see appendix C.1). In figure 3*a*–*a*”’ and *b*–*b*”’, we show the stereographic projection of a few well-chosen time frames (namely 0.5*τ*_Al_, *τ*_Al_, 3*τ*_Al_ and *T*_final_) for two of these simulations (respectively from *Movie3.mp4* and *Movie4.mp4*). They correspond to dense systems with *χ*_link_ = 0.8 and two different crosslink dynamics: fast remodelling network *ν*_link_ = 0.1 (*a*–*a*”’, *Movie3.mp4*) and slow remodelling network *ν*_link_ = 0.001 (*b*–*b*”’, *Movie4.mp4*). These screenshots enable us to answer the important question of how the network global structure emerges. It is not by accretion around a few structured areas that gradually merge together, but by an overall homogeneous structuring. Indeed, one can observe that the directional vectors gradually concentrate around a main direction without creating clustered points that merge together. This behaviour can be observed both for very aligned networks (A–A”’) or curved states (B–B”’), and in fact in all our simulations, independently on the network density. Therefore, our model suggests that the emergence of tissue architecture occurs on a global scale.

We now turn towards the analysis of the time trajectories of the quantifiers of the structures. We show in figure 3*c*, the trajectory in the phase plane A_max_ versus Al_sim_ of simulations for low-dynamical dense networks *ν*_link_ = 0.001 with various linked fibre fractions *χ*_link_ (different colours, see electronic supplementary material, appendix C.4 for more dynamical networks). We observe that all the trajectories follow a common pattern. It begins with a sharp increase of the alignment indicator (from 0.15 to between 0.4 and 0.5) while maintaining a quasi-constant semi-major axis length: this corresponds to the partial depletion of one direction (denoted *d*_1_) in the family of the fibres' directional vector, thus shifting from the initial uniform distribution to a mainly two-directional distribution (see appendix B.2 for more details on this interpretation). Non-dynamical networks do not go past that first stage (data not shown).

The trajectories then diversify: the alignment indicator keeps increasing while the semi-major axis length either decreases, stays constant or slightly increases. The first case is the most common and indicates that, while direction *d*_1_ keeps depleting until near extinction, one of the two remaining directions starts to deplete as well. This diversification happens on the scale of the time-constant *τ*_Al_ of the alignment indicator (marked on the trajectories of figure 3*c* with a black circle).

Lastly, simulations ending in an aligned state and part of those ending in a curved state display a stage of condensation of the fibres directional vectors around a main direction. This is marked by a shrinking of the covariance ellipse and a slow increase of the alignment indicator, which has already nearly reached its steady state (compare with the stabilization of Al_mean_ in figure 10). This last point comes from the local quality of the quantifier Al_sim_ (and by extension Al_mean_): a system can be very aligned locally, but not globally, if the main direction of the local structures varies smoothly across space. Thus, the transition between a curved and an aligned state is mostly characterized by a gradual shifting of multiple local structures towards the same direction.

Finally, we observe that the number of links per fibre (displayed in figure 3*d*) undergoes a transient increase followed by a two-stage exponential decay in time (appearing as a piece-wise linear decrease on the semi-logarithmic scale). For low dynamical networks, the initial accumulation of crosslinks is more pronounced, in the sense that the peak is higher and the subsequent decrease slower, when *χ*_link_ is high. For the extreme case of large linked fibre fraction *χ*_link_ = 0.9 (pink curve in figure 3*d*), the phenomenon is so strong that only the first stage of exponential decay is observed during the time of the simulation. On the other hand, for small equilibrium linked fibre fraction (*χ*_link_ = 0.1, blue curve), we do not observe any crosslinks accumulation or fast remodelling networks (see electronic supplementary material, appendix C.4 for more dynamical networks).

This behaviour can be explained by comparing the linking dynamics to the characteristic time of the repulsive interaction *T*_rep_ = 4.32 *U*_*t*_. Parameter *χ*_link_ describes the proportion of linked fibres among all linkable fibres at equilibrium, but this equilibrium takes time to establish (inversely proportional to *ν*_link_). If the repulsion interaction operates faster than the links remodelling (i.e. *T*_rep_ ≪ 1/*ν*_link_), then the linkable configurations will change before the linking/unlinking processes could equilibrate on the current configuration: new links will appear between newly overlapping fibres while former overlapping fibres will still be linked even if not overlapping anymore, leading to an accumulation of links in the system. This happens all the more if the disparity between the frequencies *ν*_link_ and *ν*_unlink_ is more favourable to linking than unlinking (*ν*_link_ > *ν*_unlink_, i.e. if *χ*_link_ > 0.5).

The system thus exhibits a global, macroscopic relaxation phenomenon which emerges from its various local, microscopic properties. It can be seen that the characteristic time-scale of this relaxation is comparable to the time-constant of the alignment indicator *τ*_Al_ (see position of the black circles on the curves in figure 3*d*, which indicates the value of *τ*_Al_ for the corresponding set of parameters).

These results demonstrate a nonlinear dependence of the network properties on the type of links and the number of crosslinks per fibre. A high number of long-lasting crosslinks promotes crosslink accumulation resulting in medium/low alignment, while fast remodelling reduces the mechanical action of the individual links on the overall network, resulting in lowly connected networks being unable to align. Together with the results of §3.2, we showed that the network alignment abilities require a number of links adapted to their remodelling speed: fast remodelling networks need a high equilibrium linked fibre fraction to quickly reach a high alignment indicator, while slow remodelling networks need a medium/low equilibrium linked fibre fraction to prevent crosslink accumulation and avoid the formation of crowded interconnected fibre structures inhibiting fibre motion.

## 4. Discussion

In this work, we have implemented a three-dimensional model for fibre networks composed of fibre elements capable of dynamically crosslinking or unlinking from each other, to align with each other at the crosslinks and to repel their nearest neighbours to prevent cluttering. We showed that this model can spontaneously generate various types of macrostructures whose emergence can be finely described. The model reveals that the different macrostructures (i) can be easily explained by a single emerging intermediate variable, namely the number of links per fibre in the ECM network, (ii) are controlled by a nonlinear relationship between the linked fibre fraction and remodelling rate and (iii) follow the same unique evolutionary path for all structures and not multiple paths.

To our knowledge, this work is the first exhaustive study questioning the mechanisms of tissue architecture emergence via a simple mechanical model of dynamical fibre networks in three dimensions. The equilibrium structures obtained with our model can be classified into three types: (a) aligned states with a strong organization around one main direction, (b) curved states with a median, locally heterogeneous alignment indicator and a wide range of directional vectors living in a plane, named curved patterns and (c) unorganized states with very low alignment indicator and no preferential direction. These different types of macro architectures show that the model can cover a wide range of biological tissues, from highly aligned fibre structures reminiscent of muscular tissues [46] to disturbed alignment of collagen fibres observed in the first phase of wound healing [47]. Unorganized states were exclusively obtained for non-dynamical networks composed of permanent crosslinks (*ν*_link_ = 0), whose plasticity was very low due to their inability to rearrange their crosslinks. By contrast, dynamical networks exhibited a mixture of aligned and curved states. These results point to the essential role of matrix remodelling in ECM structuring, consistent with several results in the literature (see [48] and references therein).

This framework reveals that the different tissue architectures at equilibrium are directly controlled by a simple intermediary variable, the number of links per fibre (see §3.1). Our interpretation is that, when the number of links per fibre is inferior to the critical threshold *N*_critic_, the network is weakly constrained. In this configuration, an increase in the number of links per fibre improves the transmission of information in the network and thus enhances the alignment process. The logarithmic scaling indicates that the higher the number of links per fibre, the less prominent this feature becomes, until the gain (in terms of the equilibrium alignment indicator) becomes null. The system then shifts into a constricted regime where each new link adds to the constriction of the network and impedes its reorganization, leading to a decrease of the local alignment.

The fact that we observe the same correlation for all dynamical networks means that, as long as a network is slightly dynamical, its final alignment is mostly controlled by its number of links per fibre rather than by its remodelling dynamics or its density. On the other hand, non-dynamical networks are locked in mechanically constrained configurations, preventing the system from reorganizing efficiently compared to dynamical ones and leading to a much lower level of alignment. However, we showed that non-dynamical networks still contain some degrees of freedom allowing for spatial matrix reorganization, and that this organization is controlled again by the number of links per fibre in the network but also by the matrix density, which becomes an important factor. Our interpretation is that dense non-dynamical networks are more spatially constrained than sparse networks. Therefore, adding new links to a sparse network can be more beneficial for the networks' overall alignment than to a dense network which has less degrees of freedom.

Altogether, this simple model suggests that different tissue architectures (different levels of fibre alignment) can already emerge as a result of simple interactions between dynamically linked fibres without the need for supplementary complex interactions involving external factors. Such simplified systems highlight the essential role of matrix remodelling on the tissue structuring. The existence of a simple emerging variable such as the number of links per fibre to control tissue structuring could have major therapeutic implications in systems where the architecture of the ECM is impacted (scarring, fibrosis, ageing), but could also prove very useful in the field of tissue engineering. Indeed, because of its simplicity, this variable is amenable to experimental measurements and represents a new putative target for the development of therapeutic drugs one could develop to restore the architecture of various biological tissues after external or internal alterations. It is noteworthy that this variable is not prescribed by model parameters but emerges from the initial simple rules as a combination of ECM remodelling dynamics, linked fibre fraction and fibre spatial organization.

The second major contribution lies in the analysis of the link between this emerging variable and the model parameters related to the crosslinks. Our model reveals that the number of links per fibre in the network, and therefore the global alignment abilities of dynamical fibre networks, results from a complex interplay between their linked fibre fraction and their remodelling speed. From such results, it is apparent that changes in linked fibre fraction will increase or decrease the global alignment abilities of the network, depending on the network remodelling rate. Thus, biological contexts in which fibre crosslinking activity undergoes changes may play an underappreciated role in driving tissue restructuring. Moreover, these results suggest that the development of biological crosslinkers controlling ECM crosslinking as a target for tissue reconstruction must be carefully accounting for ECM remodelling dynamics.

Finally, the third major contribution of the paper lies in the fine time evolution of the spatial structures. This documents the different temporal evolution of the structures as function of the ECM remodelling speeds and reveals an unique trajectory for all architectures combined with internal and transient temporal windows during which they self-organize. The model revealed that dynamical networks composed of long-lasting links exhibited a phase of crosslink accumulation followed by a long ‘relaxation’ phase (reduction of the number of links per fibre in the network) associated with a spatial reorganization of its fibres, while fast remodelling networks exhibited only the ‘relaxation’ phase. The long relaxation phase associated with slow realignment of the fibre units observed for slowly remodelling networks is reminiscent of the realignment phase observed on long time scales in later stages of wound healing [47]. The crosslink accumulation phase has been observed in different ECM networks, for instance in ageing tissues [9]. These new insights into the temporal evolution of the structures as function of the ECM remodelling properties could prove useful in the field of tissue engineering, where there is a need to design efficient biological crosslinkers [49,50].

In emerging systems, the characteristics of the final outcome cannot be predicted from the initial rules of the system and the paths from the initial interactions to the final equilibrium can be numerous and complex, corresponding to a stochastic evolution. This is not completely the case in our model because, if indeed the emerging macrostructures cannot be predicted from the initial rules and the emergence must be understood as a whole, the path is simple and unique and can be strongly predicted by an intermediate emerging variable (the number of links per fibre in the ECM). Altogether, our study suggests that the very aligned structures observed in fibrotic tissues could be mainly due to excess accumulation of crosslinks, consistent with the alterations of ECM structure observed as a consequence of increased crosslinking in lung fibrosis [51] or cancer [8], or again with previous studies on tissue-induced alignment of fibrous ECM [3,52]. Such deciphering of the emergence would open numerous perspectives for future investigations.

In this study, several simplifications were made to break the complexity of real ECM systems. For instance, the dynamical remodelling of the fibre network (random linking/unlinking of fibres) can be seen as an indirect way to account for the presence of remodelling cells. This abstract way of looking at cellular activity on the ECM enables us to study independently the effect of matrix remodelling on its structure, instead of pre-imposing some cellular dynamics. By leaving ECM linking/unlinking as free and independent parameters of the model, we are then able to study the respective importance of these parameters and the importance of matrix crosslinking on its architecture. The model not only reveals that remodelling is essential in the production of aligned fibre structures, it also suggests that ECM architecture could be mainly driven by the number of links per fibre in the matrix. Of course, in vivo experiments must be conducted to definitively validate this hypothesis and are out of the scope of this manuscript. On the modelling viewpoint, several perspectives can be considered. First, future works will be devoted to the study of the mechanical properties of these dynamical networks under tensile/compressive stress and shear and study the viscoelastic properties of the different networks [53]. Moreover, it is noteworthy that our model features networks composed of only one type of crosslink (permanent or transient with a given link-life). A natural perspective would be to study the self-organization abilities of networks composed of heterogeneous crosslinks, following the works of [36]. Moreover, our network features active crosslinks, i.e crosslinks that generate an alignment of the fibres they are attached to. As a result, our fibre networks are not subject to any external mechanical stimuli. It would be interesting to add cells having the ability to generate locally biophysical cues such as tension, stiffness and fibre production/degradation [54] and study these effects on the structure and mechanical properties of the ECM networks. In the same direction, a complete cell/fibre three-dimensional model could account for biomechanical feedback loops such as force-dependent fibre binding/unbinding [30,37].

Finally, we note that our fibre networks are reminiscent of nematic materials [55]. A fundamental difference compared to previous studies is the active nematic alignment of the rod-like elements due to the alignment torque at the (dynamical) crosslinks. As a result, contrary to mixtures of passive particles interacting via volume exclusion, the alignment ability of our material depends crucially on the crosslinking dynamics and the network configurations. In this framework, crosslink remodelling can be seen as thermal fluctuations enabling network alignment. Therefore, this simple model could serve as a basis for studying the influence of the environment in the collective motion of isotropic or anisotropic cells (bacteria) [56–58].

## Figures and Tables

**Figure 4.**
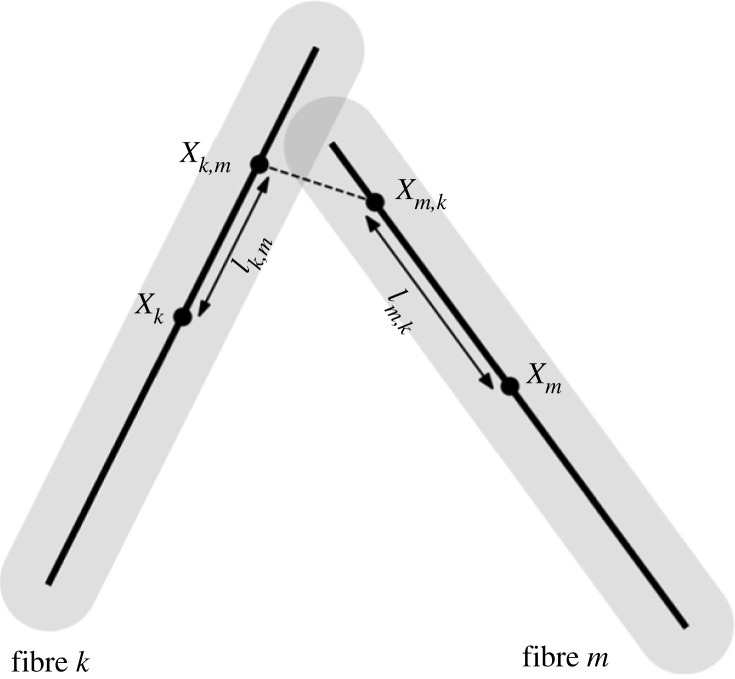
Scheme of two spherocylindrical fibres *k* and *m* indicating the position of the closest points **X**_*k*,*m*_ and **X**_*m*,*k*_ of their central segment (in a three-dimensional perspective) relative to their respective centre.

**Figure 5.**
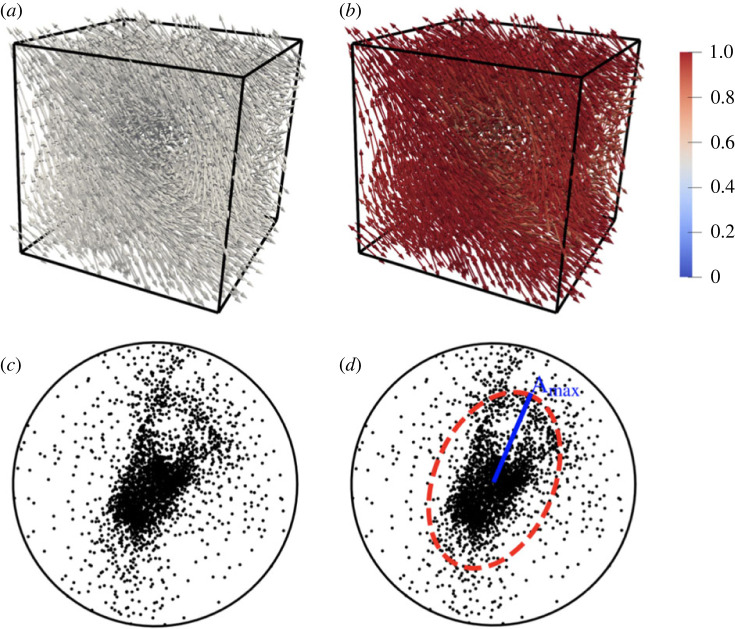
Illustration of the various way to visualize the state of a system, using as example the final state of a simulation. (*a*) Three-dimensional representation of each fibre as a grey double-headed arrow, with edges of the spatial domain Ω drawn in black. (*b*) Same representation, with fibres coloured according to their local alignment indicator (blue: Al_*k*_ = 0, red: Al_*k*_ = 1). See appendix B.1 for the actual computation. (*c*) Stereographic projection of the fibres directional vectors. See appendix B.2 for the actual computation. (*d*) Stereographic projection of the fibres directional vectors, with the covariance ellipse drawn in red dashed line and its semi-major axis drawn in blue solid line.

**Figure 6.**
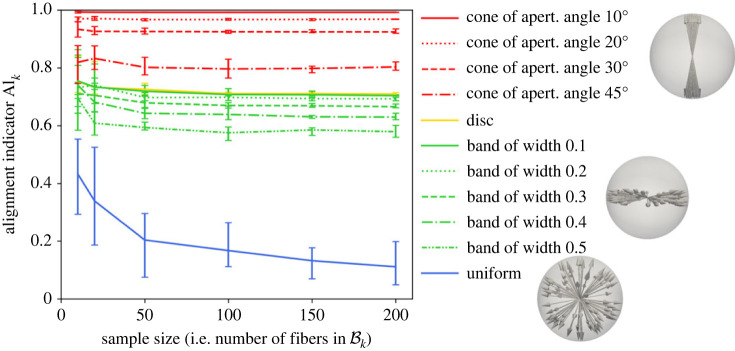
Calibration of the alignment indicator quantifier Al on random sets of orientation vectors, for various distribution laws and sample sizes. The displayed values correspond to the average and standard deviation over 10 random draws with the same characteristic.

**Figure 7.**
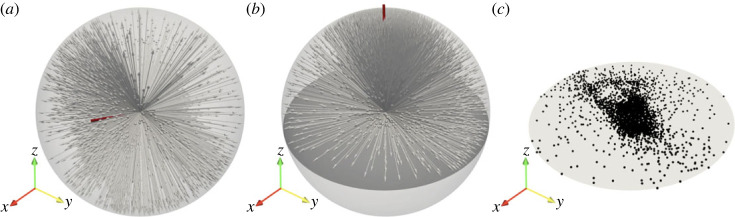
Illustration of the stereographic projection. The orientation axes are shown for reference. (*a*) Natural distribution of the fibres directional vectors on the unit sphere $S_{2}$, with main direction indicated by a red line. (*b*) Rotation of the vectors set so that its main direction (in red) now lies along the *z*-axis. The definition-space of the vectors have been reduced to the ‘North Hemisphere’, that is to the subset $S_{2}^{+}$ in the new rotated coordinates system. The equatorial plane is shown in dark grey. (*c*) Projection of the vectors onto the equatorial plane, shown in three-dimensional perspective.

## Data Availability

Data and relevant code for this research work are stored in GitHub: https://github.com/chassonnery/3D_DynamicalFiberNetwork and have been archived within the Zenodo repository: https://doi.org/10.5281/zenodo.8416498 [59]. Supplementary material is available online [60].

## Appendix A. Model

Here, we give details about the mathematical model presented in §2.1 of the main text. Let us recall the main features and introduce some notations. The *N*_fib_ fibre elements are represented by their centres $X_{k}(t)\in R^{3}$ and their non-oriented directional unit vectors $\omega_{k}(t)\in S_{2}^{+}$. The fibres repel their close neighbours by means of a soft repulsion mechanism modelling steric repulsion between spherocylinders of radius *R*_fib_. Fibre elements have the ability to link to or unlink from each other to model fibre elongation or rupture. The linking and unlinking of fibres follow random (Poisson) processes in time. Fibres offer resistance to bending through an alignment torque acting between two linked fibre elements. Fibre motion and rotation is given by Newton’s second law of motion, in an overdamped regime to model a medium with low Reynolds number.

The outline of this appendix is the following: in appendix A.1, we give the details of the mechanical interaction forces and torques acting on the fibres. In appendix A.2, we give details on the numerical implementation and appendix A.3 details the computation of the closest points of two finite segments used to compute the interactions and the crosslinks.

**A.1. Model components**

In this section, we give details of the computation of the forces and torques generated by the mechanical interactions described in §2.1 of the main text.

(*i) Computation of the fibre-fibre repulsion forces.* The force between two spherocylinders *k* and *m* is approximated by the force between two spheres of radius *R*_fib_, placed along the major axis of the fibre elements at such positions *X*_*k*,*m*_ and *X*_*m*,*k*_ that their distance is minimal (see appendix A.3 for the actual computation of this point). Denoting by $F_{k,m}^{\mathrm{rep}}$ the pairwise interaction force between the spherocylinders *k* and *m* and using Hertzian theory [39]

A1 $$F_{k,m}^{\mathrm{rep}}=\alpha_{\mathrm{rep}}{\left(2R_{\mathrm{fib}}-\|X_{k,m}-X_{m,k}\|\right)}^{3/2}\sqrt{2R_{\mathrm{fib}}}\times \frac{X_{k,m}-X_{m,k}}{\left\|X_{k,m}-X_{m,k}\right\|},$$

where *α*_rep_ is the maximal intensity of the fibre-fibre repulsion and *R*_fib_ the threshold beyond which the force field vanishes (this can be regarded as the ‘width’ of the fibre). This force is applied at point **X**_*k*,*m*_, thus inducing a rotational torque

A2 $$T_{k,m}^{\mathrm{rep}}=(X_{k,m}-X_{k})\wedge F_{k,m}^{\mathrm{rep}},$$

on fibre *k*.

(*ii) Computation of the fibre attachment forces due to crosslinks.* Fibres closer than the threshold $d_{\mathrm{link}}^{\max}$ can create a crosslink, modelled as a linear spring of stiffness *α*_rest_ and unloaded length $d_{\mathrm{link}}^{\mathrm{eq}}$ fixed to the two points of the crosslinked fibres that were closest at the time of its creation. Using Hooke’s Law, the elastic restoring force sustained by fibre *k* due to its link with fibre *m* reads

A3 $$F_{k,m}^{\mathrm{rest}}=\alpha_{\mathrm{rest}}(d_{\mathrm{link}}^{\mathrm{eq}}-\|X_{k,m}^{l}-X_{m,k}^{l}\|)\frac{X_{k,m}^{l}-X_{m,k}^{l}}{\left\|X_{k,m}^{l}-X_{m,k}^{l}\right\|},$$

where $X_{k,m}^{l}$ denotes the point of fibre *k* that was closest to fibre *m* at the time of the link creation. This force induces a rotational torque on fibre *k*

A4 $$T_{k,m}^{\mathrm{rest}}=(X_{k,m}^{l}-X_{k})\wedge F_{k,m}^{\mathrm{rest}}.$$

To ensure coherence between the different features of the model, we require that $2R_{\mathrm{fib}}\leq d_{\mathrm{link}}^{\mathrm{eq}}\leq d_{\mathrm{link}}^{\max}$.

(*iii) Computation of the linked fibre alignment forces.* It is characterized by a stiffness parameter *α*_align_ > 0 playing the role of a flexural modulus: the larger *α*_align_, the more rigid the fibre network. Given two linked fibres *k* and *m*, the torque sustained by the fibre *k* is such that, $\forall u\in R^{3}$,

A5 $$T_{k,m}^{\mathrm{align}}\wedge u=\alpha_{\mathrm{align}}\left((\omega_{k}\wedge {\tilde{\omega }}_{m})\wedge u+\frac{1-|\omega_{k}\cdot \omega_{m}|}{\|\omega_{k}\wedge \omega_{m}{\|}^{2}}(\omega_{k}\wedge {\tilde{\omega }}_{m})\wedge ((\omega_{k}\wedge {\tilde{\omega }}_{m})\wedge u)\right),$$

where ${\tilde{\omega }}_{m}=\mathrm{sign}(\omega_{k}\cdot \omega_{m})\cdot \omega_{m}$ so that there is no preferential orientation.

(*iv) Computation of fibre friction.* We assume that the friction sustained by an infinitesimal element of a fibre follows a Stokes Law with friction coefficient *μ*_fib_ [61]. The total friction force sustained by a fibre *k*, computed by integrating this law on the whole length of the fibre, is equal to

A6 $$F_{k}^{\mathrm{fric}}=-\mu_{\mathrm{fib}}L_{\mathrm{fib}}\frac{dX_{k}}{dt},$$

and the associated rotational torque is equal to

A7 $$T_{k}^{\mathrm{fric}}=-\mu_{\mathrm{fib}}L_{\mathrm{fib}}^{3}\omega_{k}\wedge \frac{d\omega_{k}}{dt}.$$

For the sake of simplicity and because our model features only one type of element, we assumed a single friction coefficient *μ*_fib_ for the fibre elements of our model. However, we note that several methods for computing the hydrodynamic properties of rigid macromolecules have been developed in the literature. For instance in [62], the authors present a systematic method for computing the frictional tensor of rigid particles modelled as assemblies of beads.

**A.2. Numerical implementation**

The differential system (2.1) is numerically solved using a discrete upwind Euler scheme, with adaptive time step. The linking and unlinking Poisson processes are updated between each time step. We assume that a pair of fibres cannot change its linking state more than once in a single time step: this is reasonable if the length of the time-step *dt* is small enough compared to the mean occurrence time 1/*ν* of the Poisson process, so we prescribe d*t* ≤ dt_link_ with

A8 $${\mathrm{dt}}_{\mathrm{link}}=\min\left(\frac{0.5}{\nu_{\mathrm{link}}},\frac{0.5}{\nu_{\mathrm{unlink}}}\right).$$

The probability for two fibres *k* and *m* to develop a crosslink between time *t*_*n*_ and time *t*_*n*+1_ = *t*_*n*_ + dt_*n*_ is then given by

A9 $$P(p_{k,m}(t_{n+1})=1\;|\;p_{k,m}(t_{n})=0\text{ and }\|X_{k,m}(t_{n})-X_{m,k}(t_{n})\|\leq d_{\mathrm{link}}^{\max})=1-e^{-\nu_{\mathrm{link}}{\mathrm{dt}}_{n}}$$

while the probability for a crosslink to break is given by

A10 $$P(p_{k,m}(t_{n+1})=0\;|\;p_{k,m}(t_{n})=1)=1-e^{-\nu_{\mathrm{unlink}}{\mathrm{dt}}_{n}}.$$

To ensure that agents do not swap position without even seeing each other, we also restrict the instantaneous translation of each fibre to half its radius *R*_fib_ and its rotation to $\arctan(0.1)\approx 6^{\circ }$. This implies the following upper limits for the time step:

A11 $$\begin{array}{cc} l & {\mathrm{dt}}_{\mathrm{trans}}(t_{n})=\underset{1\leq k\leq N_{\mathrm{fib}}}{\min}\left(0.5\frac{R_{\mathrm{fib}}}{\left|\left|\frac{dX_{k}}{dt}(t_{n})\right|\right|}\right)\\ \;\mathrm{and}\; & {\mathrm{dt}}_{\mathrm{rot}}(t_{n})=\underset{1\leq k\leq N_{\mathrm{fib}}}{\min}\left(\frac{0.1}{\left|\left|\frac{d\omega_{k}}{dt}(t_{n})\right|\right|}\right). \end{array}\}$$

Reduction of the computational cost is achieved by dividing the domain of simulation into cubes whose side-length is higher than the maximal range of the interactions: thus, interactions need only be computed for pairs of agents located in neighbouring cubes. The loops calculating the interactions are parallelized for further speeding up of the simulations.

One iteration of the Euler scheme proceeds as follows:

— Parallel computation of all forces and torques sustained by the agents at time *t*_*n*_ (right-hand part of equation (2.1)).
— Computation of the adaptive time step (equations (A 8) and (A 11))
  $${\mathrm{dt}}_{n}=\min({\mathrm{dt}}_{\mathrm{trans}}(t_{n}),{\mathrm{dt}}_{\mathrm{rot}}(t_{n}),{\mathrm{dt}}_{\mathrm{link}}).$$
— Motion of the agents to their new position
  $$X_{k}(t_{n+1})=X_{k}(t_{n})+{\mathrm{dt}}_{n}\frac{dX_{k}}{dt}(t_{n})$$
  and
  $$\omega_{k}(t_{n+1})=\omega_{k}(t_{n})+{\mathrm{dt}}_{n}\frac{d\omega_{k}}{dt}(t_{n})$$
— Account for periodic boundary conditions.
— Attribution of each agent to a simulation box.
— Parallel update of linking configuration (equations (A 9) and (A 10)).

**A.3. Closest points of two finite segments**

Given two fibres *k* and *m*, we denote by **X**_*k*,*m*_ = **X**_*k*_ + *l*_*k*,*m*_*ω*_*k*_ the point of fibre *k* closest to fibre *m* (figure 4). The couple (*l*_*k*,*m*_, *l*_*m*,*k*_) is the minimizer of the distance ‖**X**_*k*_ + *uω*_*k*_ − (**X**_*m*_ + *vω*_*m*_)‖ for (*u*, *v*) ∈ [ − (*L*_fib_/2), (*L*_fib_/2)]. If *ω*_*k*_ = *ω*_*m*_, there is an infinity of solutions of the form *v* = *u* + (**X**_*k*_ − **X**_*m*_) · *ω*_*k*_; in this case, we arbitrarily chose the solution with the smallest |*u*| value. Otherwise, there exists a unique solution whose analytical expression is

A12 $$\begin{array}{c} l_{k,m}=C_{L_{\mathrm{fib}}/2}\left(\frac{\left((\omega_{k}\cdot \omega_{m})\omega_{m}\cdot (X_{k}-X_{m})-\omega_{k}\cdot (X_{k}-X_{m})\right)}{\left(1-{\left(\omega_{k}\cdot \omega_{m}\right)}^{2}\right)}\right)\\ l_{m,k}=C_{L_{\mathrm{fib}}/2}\left(\frac{\left((\omega_{k}\cdot \omega_{m})\omega_{k}\cdot (X_{m}-X_{k})-\omega_{m}\cdot (X_{m}-X_{k})\right)}{\left(1-{\left(\omega_{k}\cdot \omega_{m}\right)}^{2}\right)}\right), \end{array}\}$$

where *C*_*a*_ denotes the cut-off function between −*a* and *a*.

## Appendix B. Quantifiers and visualization tools for the fibre structures

The goal of this section is to define quantifiers allowing to quantitatively describe the local and global organization of the fibre structures obtained with our computational model. Figure 5*a* shows a typical simulation (almost) at equilibrium, in which fibres are represented as grey double arrows. As one can observe, this simulation shows two levels of organization: a high local alignment and globally twisting, curving patterns located near the centre of the domain. In order to quantitatively describe these states, we now define appropriate numerical quantifiers.

**B.1. Local alignment indicator**

Let *R*_align_ denotes the sensing distance up to which a fibre may interact with its neighbours: in our model, it is equal to *L*_fib_ + 2*R*_fib_. For any fibre *k*, we define its neighbourhood $B_{k}$ as the set of all fibres located at a distance less than *R*_align_ and its local alignment indicator Al_*k*_ as the fractional anisotropy of the fibres' directional vectors within $B_{k}$.

It is computed as follows. We denote by *p*_*m*_ = *ω*_*m*_ ⊗ *ω*_*m*_ the projection matrix on the directional vector of fibre *m*. The mean of the projection matrices of the fibres inside $B_{k}$ is given by

B1 $$P_{k}=\frac{1}{\left|B_{k}\right|}\sum_{m_{s.t.}X_{m}\in B_{k}}p_{m},$$

where $\left|B_{k}\right|$ denotes the number of fibres in $B_{k}$.

The matrix *P*_*k*_ is symmetric positive-definite, so its three eigenvalues *λ*_1_(*P*_*k*_), *λ*_2_(*P*_*k*_) and *λ*_3_(*P*_*k*_) are real positive. The alignment indicator or fractional anisotropy in the neighbourhood $B_{k}$ is then equal to

B2 $${\mathrm{Al}}_{k}=\sqrt{\frac{3}{2}\frac{{\left(\lambda_{1}(P_{k})-\bar{\lambda }\right)}^{2}+{\left(\lambda_{2}(P_{k})-\bar{\lambda }\right)}^{2}+{\left(\lambda_{3}(P_{k})-\bar{\lambda }\right)}^{2}}{\lambda_{1}{\left(P_{k}\right)}^{2}+\lambda_{2}{\left(P_{k}\right)}^{2}+\lambda_{3}{\left(P_{k}\right)}^{2}}},$$

with $\bar{\lambda }=(\lambda_{1}(P_{k})+\lambda_{2}(P_{k})+\lambda_{3}(P_{k}))/3$ the mean of the eigenvalues.

Figure 5*b* shows the same simulation as figure 5*a*, but here the fibres have been coloured as a function of their local alignment indicator, from blue (Al_*k*_ = 0) to red (Al_*k*_ = 1). As one can see, the curved patterns are much easier to distinguish. Thus, the local alignment quantifier also serves as a visualization tool by supporting the qualitative, visual observation of locally organized states.

Note that Al_*k*_ = 1 if all the fibres in $B_{k}$ have the same directional vector. If the directional vectors are uniformly distributed then theoretically Al_*k*_ = 0, but this is not always the case. Indeed, the actual sampling of a random distribution may not be fully isotropic, especially if the number of elements in the sample is small. Figure 6 displays the value of the alignment indicator obtained for various distribution of fibres and various sample sizes: it can be seen that a uniform distribution produces alignment indicator ranging from 0.1 (when the sample size is large) to as much as 0.55 (when the sample size is small), and that there is a large discrepancy between different samples.

In our simulations, the number of neighbours of a fibre is very stable: between 20 and 25 for dense systems and between 10 and 15 for sparse systems. Non-dynamical networks display mean alignment indicators between 0.3 and 0.45 for dense systems and between 0.4 and 0.65 for sparse systems: these values are comparable to those observed in our calibration tests for a uniform distribution with similar sample size.

It can be seen from figure 6 that these biases are much smaller for non-isotropic distributions: for mainly two- or one-directional distributions, the values computed are nearly the same regardless of the sample size and the discrepancy between different samples is small. For a two-directional distribution (i.e. when the fibre directional vectors describe a disc), the eigenvalues on the mean projection matrix are theoretically *λ*_1_(*P*_*k*_) = *λ*_2_(*P*_*k*_) = 1/2 and *λ*_3_(*P*_*k*_) = 0, leading to a theoretical alignment indicator of $1/\sqrt{2}\approx 0.707$. This is very close to the value observed in our calibration tests (see yellow curve on figure 6). Nearly two-directional distributions, where the fibre directional vectors describe a ‘band’ or thick disc, give lower and lower alignment indicator as the prominence of the third direction (i.e. the band width) increases (see green curves on figure 6). Likewise, conical distributions, which are mainly one-directional, give an alignment indicator close to 1 which becomes lower and lower as the aperture angle of the cone increases (see red curves on figure 6).

**B.2. Stereographic projection**

The directional vectors of the fibres belong to the half unit sphere $S_{2}^{+}$. This subset of $R^{3}$ can be projected onto the unit disc in two dimensions using a stereographic projection, as explained below.

We define the main direction of a system as the eigenvector associated with the largest eigenvalue of its total projection matrix

B3 $$P_{\mathrm{tot}}=\frac{1}{N_{\mathrm{fib}}}\sum_{1\leq k\leq N_{\mathrm{fib}}}\omega_{k}⊗\omega_{k}.$$

If the system contains two or three equally represented directions (associated with equal eigenvalues), one of them is randomly selected.

We rotate the set of directional vectors so that this main direction lies on the *z*-axis or ‘north-south axis’. Since the fibres orientation is not relevant in our model, the set of directional vectors can be restricted to the ‘north hemisphere’ of the sphere. A point *ω* = (*x*, *y*, *z*) on this hemisphere can then be projected onto the equatorial plane via the following transformation:

B4 $$p(\omega )=\left(\frac{x}{1+z},\frac{y}{1+z}\right).$$

The whole process is illustrated in figure 7.

Figure 5*c* shows the stereographic projection of the simulation displayed in figure 5*a*,*b*. As one can observe, the dots are not uniformly distributed but densely packed at the centre of the figure, indicating the existence of a main preferential direction in the system. However, not all fibres have a directional vector close to this main direction: a non negligible number of dots are distributed all around the circle, meaning that all possible directions are represented in the system. Furthermore, the presence of a ‘circular branch’ in the top-right part of the point cloud allows us to identify the locally twisting structure that can be observed in figure 5*b*: in this part of the system, nearby fibres have similar but gradually shifting directional vectors such that, on the scale of the whole structure, the fibres' directional vectors describe a circle (in the domain $S_{2}^{+}$).

Thus, this representation enables us to quickly grasp the distribution of the fibres directional vectors around one or more poles. It must be noted that proximity on the stereographic projection indicates similar directional vectors, but not necessarily spatial proximity. Nonetheless, we can gain insights into the overall architecture of the network by drawing the covariance ellipse of the point-cloud (in red dashed line on figure 5*c*) and computing its semi-major axis length A_max_. As shown in the §3.2, this enables us to identify many type of ‘states’ or structures that can also hint on the spatial organization of the network.
